# The Sufferings of Christ

**Published:** 1888-04-07

**Authors:** 


### Words of Consolation.

[Communications for this column should be addressed "Chaplain," care of the Editor of The Hospital, who will gratefully welcome any suggestions or inquiries from matrons, nurses, and the staff generally.]

### LXXII.—The Sufferings of Christ.

*For Reading to the Sick.*

Besides tasting, as it were, of the second death in the desolation of the Cross, our blessed Lord also parted with His life in the usually-accepted sense. There was a separation of the soul from the body. Therefore, in the ordinary sense, His very life was sacrificed. If the full satisfaction were rendered in His perfect obedience under suffering, we must look yet further for the most essential reason for His dissolution. To understand this we will take His own illustration from the corn seed: "Verily, verily, I say unto you, Except a corn of wheat fall into the ground and die, it abideth alone: but if it die, it bringeth forth much fruit" (John xii. 24). The law of Nature proclaims that the dissolution of the seed is first essential before the new life contained therein can spring forth. Certain elements in the seed, or on its outside, have to be thrown off. It has to part with its old life in the shape of husk. Then gradually the stalk finds its way through the surface of the earth, and from the stalk the fruit is developed. But, observe, this new life is not confined to the seed itself; it becomes diffusive; it becomes many other seeds, which in turn become the security for the preservation of life in ourselves. The corn of wheat falls into the ground, is dissolved, and through dissolution becomes the multiplied life-sustaining power in bread. So you see the very object of this death in Nature is, not only that a higher life shall be given to the seed, but that the essence and principle of such life be transferred to mankind. A great mystery. But if you admit this fact in the realm of Nature, you have the true key to one of the greatest mysteries in the spiritual kingdom. It was just as essential for Christ to die, yea, for Him to submit to dissolution, in order that the higher and spiritual life centred in His humanity should be developed in and from His person. And for what purpose? Was this in order that He alone should be glorified, or that He alone should be invested with the splendid capacities of the resurrection body? No: follow the law of the seed, and you will undertand that He submitted to dissolution with a view to the principle of life within Him becoming diffusive. In other words, His life was poured forth that, becoming yet further spiritualised, it might be made the healing power, the feeding power, and at last the resurrection power in His people. This is sometimes called the extension of the Incarnation. We shall see further on how we are each, if we choose, made partakers of these fruits of His death and resurrection. Meanwhile, it will be sufficient to bear in mind how often this great truth is either obscured or very insufficiently taught by those who would have us rely so entirely on the propitiatory and atoning work of Christ, as to make believe that spiritual life can be, as it were, given and sustained continually by assenting to the fact of Christ's death upon the cross. The two truths should be kept distinct, although one grows out of the other. Faith in the sacrifice offered for sin is a thing distinct from the feeding upon the fruits of that sacrifice. The one must lead to the other. But believing in Christ and feeding upon Him are not the same thing. We are said to feed upon Him by faith, because faith leads to the act whereby we partake of the spiritual food.

